# Continued Expression of GATA3 Is Necessary for Cochlear Neurosensory Development

**DOI:** 10.1371/journal.pone.0062046

**Published:** 2013-04-16

**Authors:** Jeremy S. Duncan, Bernd Fritzsch

**Affiliations:** Department of Biology, University of Iowa, Iowa City, Iowa, United States of America; University of Washington, United States of America

## Abstract

Hair cells of the developing mammalian inner ear are progressively defined through cell fate restriction. This process culminates in the expression of the bHLH transcription factor *Atoh1*, which is necessary for differentiation of hair cells, but not for their specification. Loss of several genes will disrupt ear morphogenesis or arrest of neurosensory epithelia development. We previously showed in null mutants that the loss of the transcription factor, *Gata3*, results specifically in the loss of all cochlear neurosensory development. Temporal expression of *Gata3* is broad from the otic placode stage through the postnatal ear. It therefore remains unclear at which stage in development Gata3 exerts its effect. To better understand the stage specific effects of Gata3, we investigated the role of Gata3 in cochlear neurosensory specification and differentiation utilizing a LoxP targeted *Gata3* line and two Cre lines. *Foxg1^Cre^∶Gata3^f/f^* mice show recombination of *Gata3* around E8.5 but continue to develop a cochlear duct without differentiated hair cells and spiral ganglion neurons. qRT-PCR data show that *Atoh1* was down-regulated but not absent in the duct whereas other hair cell specific genes such as *Pou4f3* were completely absent. In addition, while *Sox2* levels were lower in the *Foxg1^Cre^:Gata3^f/f^* cochlea, *Eya1* levels remained normal. We conclude that Eya1 is unable to fully upregulate *Atoh1 or Pou4f3*, and drive differentiation of hair cells without Gata3. *Pax2-Cre∶Gata3^f/f^* mice show a delayed recombination of *Gata3* in the ear relative to *Foxg1^Cre^:Gata3^f/f^*. These mice exhibited a cochlear duct containing patches of partially differentiated hair cells and developed only few and incorrectly projecting spiral ganglion neurons. Our conditional deletion studies reveal a major role of Gata3 in the signaling of prosensory genes and in the differentiation of cochlear neurosenory cells. We suggest that Gata3 may act in combination with Eya1, Six1, and Sox2 in cochlear prosensory gene signaling.

## Introduction

The mammalian inner ear develops through a cascade of diffusible factors and transcription factors to generate a complex labyrinth of ducts and recesses out of a flat epithelium, the otic placode [1], [2]. In one of these recesses, the cochlear duct, the organ of Corti differentiates to produce a highly ordered distribution of hair cells and supporting cells whose stereotyped organization and polarization is crucial for normal hearing function. Additionally, spiral ganglion neurons connect these hair cells to the cochlear nuclei of the hindbrain. All three of these neurosensory cell types, along with non-neurosensory otic cells, are derived from a multipotent pool of otic progenitor cells [3]. As development progresses the nonsensory region is segregated from the pro-neurosensory domain [4]–[6]. While the timing of prosensory establishment is unclear, the restricted expression of *Jagged1 (Jag1), Lunatic fringe (Lfng), Bone morphogenetic protein (Bmp4,) SRY-box containing gene (Sox2), Paired box gene 2 (Pax2)* and several *Fibroblast growth factors (Fgf3, 8, 10, 20)* in the prosensory areas of the mouse at E9.5–10.5 suggests that prosensory areas are molecularly defined by these factors at or before this time point [7]–[15]. To differentiate hair cells within these prosensory areas requires precise topology, levels and duration of *Atoh1* expression [16], [17]. However, *Atoh1* is not necessary for specification of postmitotic precursors [18]–[21] which exit the cell cycle in the apex several days before Atoh1 can be demonstrated by tissue based techniques [22]. Attempts to restore lost hair cells through over-expression of *Atoh1* in the cochlea results in transformation of some cells into hair cell-like cells, but these hair cells are unstable and are morphologically and physiologically like vestibular hair cells [23]–[26]. Thus the efficiency and efficacy of *Atoh1* transfection alone is inadequate for restoration of lost hair cells in the mammalian cochlea suggesting that *Atoh1* action depends on the proper molecular context to develop organ of Corti-like hair cells as previously suggested for placodal development [1]. Indeed, data on Eya1/Six1 suggest that expression of inner ear neurosensory bHLH genes depend on other factors that cooperate in the promoter region to ultimately activate and sustain relevant genes for hair cell and neuronal differentiation [27], [28]. How many of these essential factors for bHLH gene expression are needed for the expression of bHLH factors to ensure complete and lasting hair cell differentiation in development, or to ensure replacement of lost hair cells to cure deafness, remains unclear [16]. We explore here one of these essential factors for the mammalian cochlear neurosensory development, Gata3.

The zinc finger transcription factor Gata3 is expressed throughout the early otic placode. Later Gata3 becomes restricted to future proneurosensory regions (except that of the saccule) concomitant with their segregation from non-sensory domains [29]–[31]. Expression of *Gata3* is particularly high in the ventral otocyst, the area of the cochlear anlage [30], [31]. *Gata3* expression continues in the organ of Corti and spiral ganglion neurons from specification through late postnatal stages [17], [32]–[34]. However, levels of Gata3 protein expression in the hair cells of the organ of Corti seems to decrease over time, [35] but remains in the supporting cells [17], [34].

Haploinsufficiency of GATA3 causes human hypoparathyroidism, sensorineural deafness, and renal dysplasia (HDR) syndrome [36]. While patients have different combinations of the three central phenotypes which characterize HDR syndrome, all patients studied thus far have some form of sensorineural deafness [37], indicating GATA3 has a particularly profound role in cochlear neurosensory development. Despite its apparent absolute necessity for early neurosensory formation in the cochlea [38], [39], the role of Gata3 in cochlear neurosensory specification, differentiation, and maintenance as well as the relationship of Gata3 to other pro-neurosensory genes and their cascades remains unclear due to limited viability of the mouse *Gata3* null mutant. Moreover, what specific function the later expression of Gata3 has in these processes remains unclear as no inner ear-specific delayed deletion data exist for Gata3.

We investigated the function of Gata3 at later embryonic stages utilizing two conditional *Gata3* deletion mouse models. We utilized both *Foxg1^Cre^* and *Pax2-Cre* to recombine floxed *Gata3* in the ear. *Foxg1^Cre^* is a knockin Cre that recapitulates the *Foxg1* expression from the E8–8.5 otic placode [40], [41] to the late postnatal ear [42]. In contrast, *Pax2-Cre* is a BAC-transgene that does not fully conform to early *Pax2* expression [12] and is expressed only after E9.5 [43]. It is important that recombination is determined by onset of the Cre expression in the early or late placode, respectively, and not by the late expression in the ear that sees a progressive segregation of Pax2 and Gata3 expression [31] but not of the Foxg1/Gata3 expression [42].

Our results show that *Foxg1^Cre^:Gata3^f/f^* mice have an earlier and more profound loss of *Gata3* expression and exhibit a cochlear duct completely devoid of neurosensory cells. *Atoh1* shows limited expression levels in the cochlea of these mice, suggesting that a specific *Atoh1* level is critical for hair cell differentiation. Some prosensory genes such as *Sox2* are expressed at lower levels compared to normal, while *Eya1* and others are not altered in their expression levels. This suggests that Gata3 is an essential co-factor for neurosensory development. Utilizing *Pax2-Cre: Gata3^f/f^* mice resulted in a delayed deletion of *Gata3* before differentiation of the organ of Corti. Only patches of the organ of Corti with abnormal hair cells formed in the cochlear duct along with improperly projecting spiral ganglion neurons. This indicates sustained expression of Gata3 is required to fully differentiate hair cells and spiral ganglion neurons of the organ of Corti.

## Methods

All animal care and procedures were approved by the University of Iowa IACUC following the guidelines for use of laboratory animals (ACURF 0804066).

### Mouse model and genotyping

To generate *Gata3* conditional knockout mice, we crossed *Foxg1^Cre^* mice [40] or *Pax2-Cre* [43] to *Gata3* floxed mice [44]. Nomenclature is based upon International Committee on Standardized Genetic Nomenclature for Mice, indicating the knockin Cre into the *Foxg1* locus as *Foxg1^Cre^* and *Pax2* promoter with the Cre recombinase inserted as a BAC transgene as *Pax2-Cre*. Breedings were always conducted with a male expressing Cre and heterozygous for the *Gata3* floxed allele and the female being homozygous for the floxed allele and no Cre. A few of the matings included a *Pax2-Cre* male with a *Gata3* LacZ null allele instead of a *Gata3* floxed allele [45]. Instances where this occurred are noted in the text and figures. There were no noticeable differences between the *Pax2-Cre:Gata3^LacZ/f^* and *Pax2-Cre:Gata3^f/f^* ears. Genotyping by PCR analysis of tail DNA was accomplished under standard conditions with *Gata3* specific primers (*Gata3* forward; 5′-GATTCAGTCTCCCTCCTTCTTC-3′; *Gata3* reverse 5′GTTCACACACTCCCTGCCTTCTG-3′). Embryos were collected from pregnant dams with noon on the day of vaginal plug considered embryonic day (E) 0.5. To collect embryos, pregnant dams were anesthetized with 1.25% Avertin at a dose of 0.025 ml/g by intraperitoneal injection. For non-qRT-PCR experiments, embryos were dissected from the uterus and fixed with 4% paraformaldehyde (PFA) in 0.1 M phosphate buffer (pH 7.4) transcardially using a peristaltic pump. The following genotypes were utilized as controls: *Pax2-Cre*:*Gata3* ^wt/wt^; *Foxg1^Cre^*:*Gata3* ^wt/wt^; *Gata3* ^f/wt^; *Gata3* ^f/f^. All images are representative of assessments of at least 3 biological controls which were identical unless noted in the text.

### *In situ* hybridization

Whole-mount *in situ* hybridization experiments were conducted as described in [46]. Briefly, control and conditional knock-out mice were fixed in 4% PFA and dissected in 0.4% PFA RNAse-free conditions. From this point forward a control and conditional knock-out were always run in the same vial (one right earand one left ear) to expose mutant and control to the same experimental conditions throughout. Ears were defatted in 100% methanol and rehydrated through a graded methanol series, digested with proteinase K (Ambion), and then hybridized to a specific riboprobe overnight at 60°C. Unbound probe was washed off and the tissue was incubated overnight with anti-digoxigenin antibody (Roche) conjugated with alkaline phosphatase at room temperature. The probe was detected using BM Purple AP substrate (Roche). The tissue was mounted in glycerol and imaged with a Nikon E800 microscope and images were captured using MetaMorph software (Universal Imaging Corporation). Care was taken to insure microscope and software settings were identical for paired samples and images.

### Immunochemistry

Whole-mount Immunochemistry was performed as described in [46]. Briefly, ears were dissected and control and conditional knock-out ears were always run in the same vial (one right ear, one left ear to differentiate). Ears were defatted in 75% ethanol overnight, and rehydrated through a graded ethanol series. Blocking was performed in 2.5% normal goat serum and 0.5% Triton X-100 at room temperature on a shaker. Samples were rinsed in PBS for one hour then placed in blocking solution and one or a combination of the primary antibodies for Myo7a (1∶200; Proteus Biosciences), anti-acetylated α-Tubulin (1∶800; Sigma), Sox2 (1∶500; Millipore), Bdnf (1∶500; Invitrogen). The samples were then incubated for 48 hours at 4°C. Samples were then rinsed three times with PBS over one hour and placed in blocking solution for one hour. Secondary antibodies (Alexa flour 488, 532, 633; Invitrogen) were added at a 1∶500 dilution in block solution for 24 hours at 4°C on a shaker. Samples were mounted on a glass slide in glycerol and images were taken with a Leica TCS SP5 confocal microscope. Images were compiled using CorelDraw ×5.

### Lipophilic dye tracing

Inner ear innervation was labeled with lipophylic (NeuroVue) dyes (Molecular Targeting Technologies; MTTI). Dyes were placed into the cerebellum and caudal hindbrain for selective labeling of vestibulo-cerebellar and vestibulo-spinal nerves as described [47], [48]. Dye was placed in the contralateral or ipsilateral rhombomere 4 for pure contralateral vestibulo-cochlear efferent or combined vestibulo-cochlear efferent and facial nerve respectively [49]. Dye was also placed into the ear to label vestibulo-cochlear efferent, facial, and vestibulo-cochlear afferent projections to the brainstem. The preparations were then incubated in 4% PFA at 60°C for two to four days depending on the age of the embryos. After dye diffusion either the hindbrain or ear was dissected out and placed on a glass slide with glycerol and a cover slip on top to be imaged with a Leica SP5 confocal microscope.

### qRT-PCR

Primers were created using the Roche Universal Probe library assay center which utilizes Advance Primer3 to calculate optimal size, melting point, and complementarity of primer sets. A list of primers used can be found in Table S1. Hydrolysis probes were matched with primer sets and chosen according to the same Universal Probe Library assay center. RNA was extracted using the RNeasy Mini Kit (Qiagen; Valencia, CA). RNA was then reverse transcribed with Super Script III first strand synthesis kit (Invitrogen). Primers, probes, cDNA, Roche Probe Master Mix, and water were then combined into a 96 well PCR plate according to the Probe Master Mix directions. qRT-PCR was performed using a Roche Lightcycler 480 according to the Probe Master Mix instructions. Data was then analyzed using the Lightcycler 480 software version 1.5. All primer pairs and probes were verified both by conducting serial dilutions of RNA before reverse transcription, to asses reverse transcription inhibition, and cDNA dilution curves. Actb was used as a reference gene when comparing mutant and control. All procedures followed the MIQE protocol of triplicate biological and technical repeats [50].

### 3D reconstruction

The process of staining, imaging, and reconstruction of inner ears has been previously described [51]. Ears were dissected from surrounding tissue and dehydrated with an increasing ethanol series ending with 100% ethanol for 12 hours. Ears were then stained with a solution of Rhodamine B isothiocyanate 1 mg/200 ml of 100% ethanol; staining all tissue. Subsequently the ears were cleared with Spalteholz solution (5∶3 methyl salicylate:benzyl benzoate). A glass slide in which a ring of vacuum grease (Dow Corning) was created to contain a pool of Spalteholz solution. Ears were placed in this pool and a cover slip was placed on top. The thickness of the vacuum grease was greater than the thickness of the ear to avoid compression of the ear. Specimens were imaged on a Leica SP5 confocal microscope. The image stack for each ear was loaded into Amira software (Visage Imaging, San Diego, CA.) for 3D reconstruction [52]. To isolate the different inner ear structures, segmentation was performed using Amira's segmentation function. Endolymphatic and perilymphatic spaces being devoid of tissue were clearly identified from the rhodamine stained surrounding tissue. These spaces were segmented leading to the 3D reconstruction. After segmentation, Amira calculated the surface volume and reconstructed the 3D shape of the inner ear.

### Scanning Electron Microscopy (SEM)

The mice for scanning electron microscopy were perfused with 4% PFA after sedating with Avertin. Ears were then dissected and fixed in a 2.5% glutaraldehyde, 4% PFA solution. Ears of postnatal mice were decalcified with EDTA. Following osmication in 2% osmium tetroxide in 0.1 Monophosphate buffer (pH 7.4) for 1 hour, the ears were dissected and prepared including removal of the tectorial membrane. The samples were then rinsed with distilled water to remove ions and dehydrated in a graded ethanol series, critical-point dried, mounted on stubs with carbon tape and coated with gold/palladium. Specimens were then viewed with a Hitachi S-4800 Scanning Electron Microscope with 3MeV acceleration. False coloring was accomplished with Adobe Photoshop CS6.

## Results

### *Foxg1^Cre^∶ Gata3^f/f^* ears lack cochlear neurosensory epithelia

**Figure 1:**
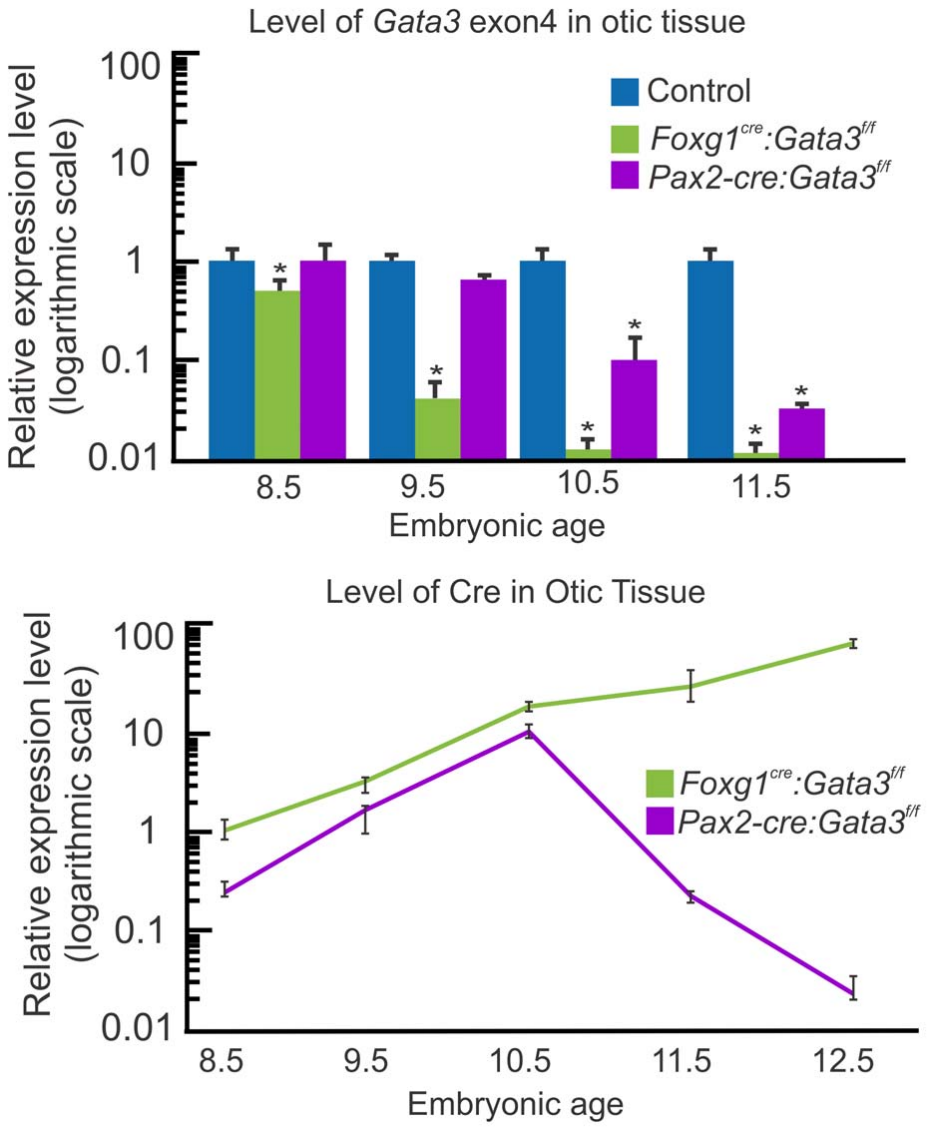
*Gata3* recombination. The top panel, using qRT-PCR, shows the relative level of recombined *Gata3* exon4 in the inner ear of both Cre lines compared to control (set to 1, for each age group). There is an earlier and more effective recombination of the *Gata3* floxed allele in *Foxg1^Cre^* ears than in *Pax2-Cre*. *Pax2-Cre* ears first show a significant reduction in *Gata3* exon4 at E10.5 two days after *Foxg1^Cre^*. This is correlated with the timing of sensory vs non-sensory specification in the ear. The lower panel shows a comparison of Cre expression levels, using qRT-PCR, between *Foxg1^Cre^: Gata3^f/f^* and *Pax2-Cre:Gata3^f/f^* ears. Cre expression levels are relative to the level of Cre mRNA in *Foxg1^Cre^: Gata3f/f* ears at E8.5 (set to 1). The scale on the left is logarithmic. *Pax2-Cre* mRNA expression levels are 8-fold less at E8.5 than *Foxg1^Cre^*. All data represent 3 biological replicates and 3 technical replicates per-time point as per MIQE guidelines [50]. *p<.01, students t-test.

Previously we have reported that *Gata3* null ears fail to develop all inner ear neurosensory epithelia except a few saccular hair cells and neurons [38]. Variable success rates in rescuing mutants combined with variable effects proved problematic for further analysis. To bypass this issue, we combined a conditional knock-out *Gata3* mouse line [53] with Foxg1^Cre^ [40]. The floxed *Gata3* line has LoxP sites flanking exon4 which contains the primary DNA binding domain. *Foxg1^Cre^* is expressed early in ear development [40], but not in the hindbrain where loss of *Gata3* is proposed to lead to early embryonic lethality. To assess recombination of the floxed allele by Foxg1^Cre^ we used qRT-PCR to assess *Gata3* mRNA levels over time. We designed specific *Gata3* exon4 primers to detect recombination. *Gata3* exon4 mRNA expression was significantly reduced in mutant ears by embryonic day (E) 8.5 compared to control, and continued to decrease with age (Fig. 1). Level of Cre was also assessed in *Foxg1Cre: Gata3^f/f^* ears over time, and from E8.5-E12.5 *Cre* levels continued to rise in the ear (Fig. 1).

**Figure 2:**
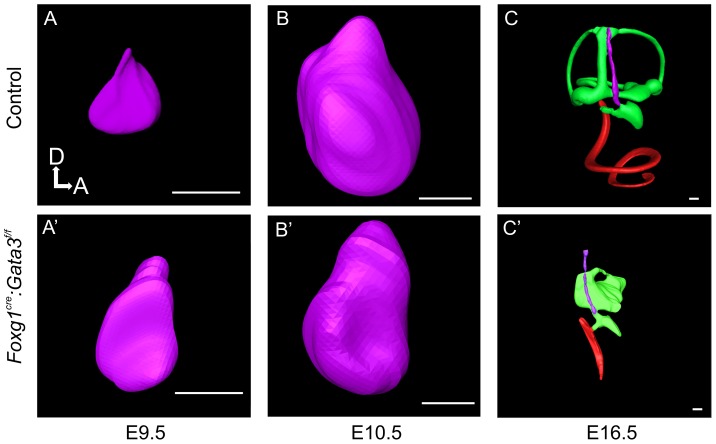
Morphological development of *Foxg1^Cre^∶ Gata3^f/f^* ears. 3D reconstruction of *Foxg1^Cre^*:*Gata3^f/f^* inner ears compared to control; utilizing confocal microscopy and Amira software. **A-C**) At E9.5 there is very little difference in size between the two genotypes. By E10.5 there is a slight reduction in the mutant ear dorsally. At E16.5 there is morphologic development of a cochlear duct (red) in the mutant ear, although it is truncated compared to control. There is also morphologic development of the vestibular portion of the mutant ear (green) although it is highly abnormal. While there is a noticeable saccular out-pouching, none of the other vestibular structures are easily identifiable. The endolymphatic duct is present in the mutant ear (purple) and extends dorsally. Dorsal is up anterior is to the right in all images. All scale bars represent 100 µm.

We next performed confocal 3D reconstructions to determine the morphologic phenotype of *Foxg1^Cre^: Gata3^f/f^* ears compared to control. At E9.5 and E10.5 there were no obvious differences in size or overall morphology of the otic vesicle. At E16.5 the overall size of the *Foxg1^Cre^: Gata3^f/f^* ear was drastically reduced compared to the same stage control ear (Fig. 2). In addition to the overall size reduction, the vestibular semicircular canals failed to develop. The shape of the mutant ear showed very limited medio-lateral expansion after E10.5 compared to the control ear. The cochlear duct length was severely truncated and showed virtually no coiling (Fig. 2). Both the non-sensory endolymphatic duct and the saccular out-pouching areas lacking *Gata3* expression during normal development were also reduced in these mutants. This striking morphology was present in all mutants analyzed with no noticeable variability. The morphologic features of the *Foxg1^Cre^:Gata3^f/f^* cochlea were in line with the full null phenotype, but displayed a more developed vestibular portion of the ear [38], [39].

**Figure 3:**
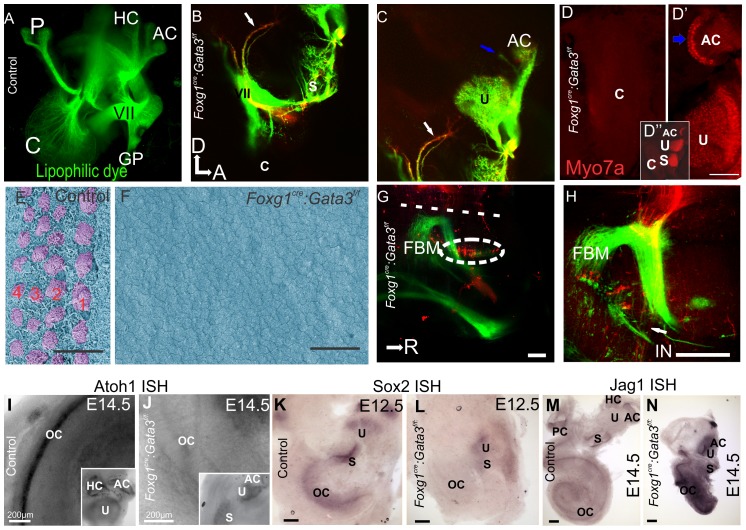
*Foxg1^Cre^*∶*Gata3^f/f^* mice show loss of cochlear neurosensory epithelia. **A**) E16.5 Control ear showing lipophilic dye tracing of normal wild-type distribution of efferent, VII, and GP nerves. A portion of the posterior projection of the VII nerve was removed in order to visualize the cochlear (C) afferents. Injection of dye was in ipsilateral rhombomere 4. **B,C**) Injection of lipophilic dye in E16.5 *Foxg1^Cre^*:*Gata3^f/f^* hindbrain to label fibers to the ear as in (**A**), except in addition red lipophilic dye was injected to label the cochlear and vestibular afferents. The facial nerve (VII) shows a normal course around the ear. No fibers are projecting towards the cochlear duct (c). Blue arrow indicates fibers projecting to a non-existent horizontal canal. White arrow indicates fibers initially projecting to the location of where a posterior canal should reside, but in its absence these fibers diverge and project to the remaining epithelia. **D**) *Foxg1^Cre^*: *Gata3^f/f^* ear labeled with Myo7a immunohistochemistry shown in red. Positive cells are located in the saccule, utricle, and one canal crista. There are no Myo7a positive cells in the cochlear duct. In the center of the single canal crista there is a lack of non-sensory epithelia (blue arrow). **D**′′) Low magnification of entire mutant ear. **E**) SEM of E16.5 control organ of Corti, showing the normal stereotyped pattern of four rows of hair cells (lilac). The early tectorial membrane could be visualized during preparation. **F**) SEM showing a flat epithelium in the *Foxg1^Cre^*: *Gata3^f/f^* ear. No hair cells could be identified in the cochlear duct. No tectorial membrane was observed during dissection. There was also a lack of microvilli on the remaining epithelia. **G,H**) Inner ear efferents and FBM were both labeled with red dye on the same side simultaneously where the facial nerve wraps around the ear. The contralateral FBM were labeled with green dye. **G**) Efferent cell bodies can be seen (as marked by the white oval). These cells were labeled from the contralateral ear. **H**) Efferent axons can be seen (arrow) as they diverge from the facial nerve. All aspects of efferent cell body location are in concordance with wild type phenotype. **I and J**) Showing *Atoh1* in situ hybridization (ISH) at E14.5. Insets show positive expression remains in the vestibular portion of the ear correlating with the presence of Myo7a positive cells, and absence within the mutant cochlear duct. **K and L**) Showing Sox2 ISH at e12.5. Sox2 is necessary for specification of sensory epithelia in the ear. In the control ear (**K**) all sensory epithelia show positive expression. In the Foxg1^Cre^: *Gata3^f/f^* ear (**L**) the vestibular portion is positive for Sox2 expression while the organ of Corti shows no expression. **M and N**) Showing Jag1 ISH at E14.5. Jag1 is a known marker of the prosensory domain and necessary for sensory cell development in the inner ear. Jag1 expression appears more highly upregulated with respect to topology and intensity in the mutant (**N**) compared to control (**M**). Anterior is to the right and dorsal is up in all images. Scale bars C and D indicate 10 µm; G,H, K-N indicate 100 µm, I and J indicate 200 µm. A-F, dorsal is up and anterior is to the right. G,H rostral is to the right. c, cochlea; p, posterior crista; hc, horizontal crista; ac, anterior crista; VII, facial nerve; gp, greater petrosal nerve; u, uturicle; s, saccule; FBM, facial branchial motorneurons; IN, intermediate nerve; oc, organ of Corti.

To examine the neuronal phenotype, two different fluorescing lipophilic dyes were placed in the cochlear/vestibular nuclei labeling all inner ear afferents and in rhombomere 4 labeling inner ear efferents, as previously reported [54]. The utricle, saccule, and a single canal crista were innervated by both afferent and efferent fibers revealed with differential injection of lipophilic dyes. Projections destined for the posterior canal crista reached the area where the crista should be located, however, many of these fibers turned and projected towards the remaining sensory epithelia of the anterior ear (Fig. 3A-C). There were also projections to the area of the ear in which a horizontal canal should have formed (Fig. 3C). However, we never detected hair cells positive for Myo7a in this area (Fig. 3D′′, see below). Most notably, no cochlear neurons could be visualized in any mutants assessed at any stage (n = 7, Fig. 3B). These data indicate that some vestibular neurons form and can project somewhat stereotypically across the ear, even if no target in terms of sensory epithelia exist, complimentary with previous data [11]. However, early loss of Gata3 is incompatible with spiral ganglion cell formation.

To determine the extent of sensory epithelia development, hair cells were visualized by Myo7a immunochemistry, a specific marker for hair cells (Fig. 3D). Instead of six discrete patches of hair cells identifiable in the control animals, mutants showed only three patches of hair cells. These data suggest that the mutant cochlear duct completely lacked hair cells (Fig. 3D). In contrast, the vestibular system was reduced to a utricle, saccule, and a single canal crista identifiable based on their topography, Myo7a positive hair cell distribution, and pattern of innervation. The single canal crista, although containing hair cells, lacked normal morphologic characteristics such as the non-sensory septum cruciatum (Fig. 3D′), which normally divides the sensory area into two halves, and normally expresses *Gata3* [30]. The other two canal cristae were absent (Fig. 3D′′).

SEM analysis confirmed the lack of histogenesis of the *Foxg1^Cre^: Gata3^f/f^* cochlea. In the control cochlea there are three rows of outer and one row of inner hair cells (Fig. 3E). Consistent with the lack of Myo7a positive cells, in the *Foxg1^Cre^: Gata3^f/f^* mutant there were no cells with typical early hair cell morphology and there was an absence of microvilli on the surrounding cells (Fig. 3F). Early loss of Gata3 results in a cochlear duct with a completely flat epithelium as seen in mutants for *Atoh1* [17], [18], [20]. However, in contrast to the normal extension of the cochlear duct in Atoh1 null mice [17], [20], the cochlear duct is drastically reduced in Foxg1^Cre^ conditional mutant mice. There was also a complete lack of tectorial membrane formation revealing a simple epithelial cochlear duct wall.

In the absence of the organ of Corti the olivo-cochlear efferents lack a peripheral target and we showed above that no neuronal projections reach the cochlear duct (Fig. 3). We placed distinctly colored lipophilic dyes in both ears to label the afferent and efferent neurons, and assessed their topology within the hindbrain. In doing so we also labeled the nearby facial nerve to secure proper diffusion distance and coverage. Even though cochlear efferents in the mutant did not project to the cochlea, we labelled olivo-cochlear neurons from the ear/facial nerve and could show their normal topological distribution within the hindbrain (Fig. 3G,H). Thus, while cochlear efferent fibers did not project peripherally to the correct target, the olivo-cochlear efferent soma migrated into the correct periolivary region [49]. Because we were able to label these olivocochlear efferents with application of the dye to the inner ear they are most likely either projecting to vestibular sensory epithelia, or are projecting with the facial nerve as described in *Gata3* null mutants [38] and Neurog1 null mutants [55].

In summary, *Foxg1^Cre^: Gata3^f/f^* lack any neurosensory differentiation of the organ of Corti but form a shortened cochlear duct and develop olivo-cochlear neurons with peripheral projections to non-cochlea targets.

### Gene expression in *Foxg1^Cre^: Gata3^f/f^* ears

We used *in situ* hybridization against known genes in the specification and differentiation of cell types in the cochlea to assess spatial expression of genes in the absence of *Gata3* (Fig. 3I-N). *Atoh1*, a gene necessary for hair cell differentiation, was undetectable by ISH in the *Foxg1^Cre^: Gata3^f/f^* cochlea. However, *Atoh1* expression remained in the vestibular portions where hair cells formed (Fig. 3J). *Sox2*, a gene necessary for prosensory development, was expressed in control embryos throughout the cochlear duct, and all vestibular sensory epithelia (Fig. 3K). In the *Foxg1^Cre^: Gata3^f/f^* mutant, *Sox2* expression could be seen in the utricle and saccule but not in the cochlear duct (Fig. 3L). *Jag1*, is a membrane based ligand of the delta/notch signaling system, and mutants for this gene show loss of most cochlear sensory epithelia [56]. Additionally, *Jag1* has been shown to be necessary for maintenance of *Sox2* expression, but is unable to upregulate *Sox2* in areas previously devoid of *Sox2* expression [57], [58]. In the mutant cochlea, *Jag1* was upregulated both topologically and in intensity (Fig. 3N). This robust *Jag1* expression indicates that even in the presence of *Jag1* prosensory cells are unable to fully express other prosensory markers such as *Sox2* and cannot differentiate hair cells without Gata3.

**Figure 4:**
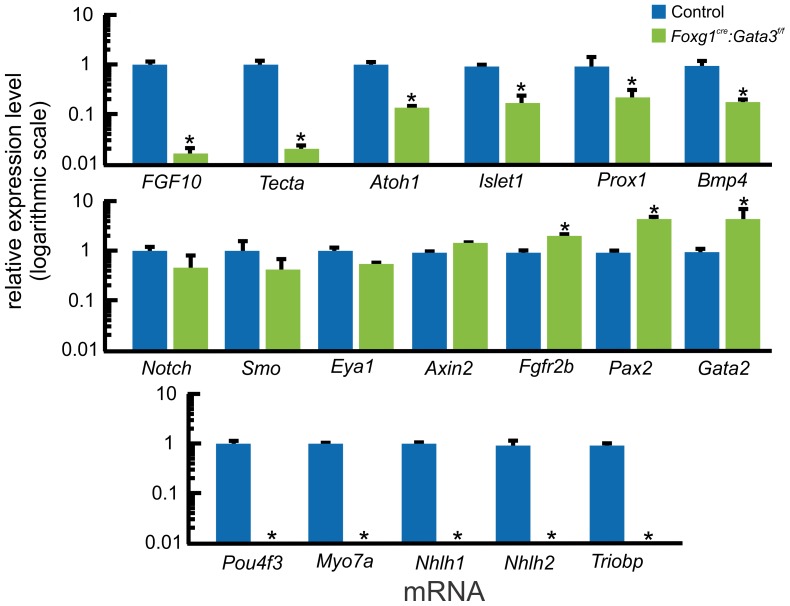
Many genes known in cochlear neurosensory development show altered expression levels in the absence of *Gata3*. **A**) qRT-PCR analysis comparing gene expression of E16.5 control (set to1) and Foxg1^Cre^: *Gata3^f/f^* microdissected cochleas. Gene comparisons were normalized with *Actb*. *Fgf10* has previously been shown to be regulated by Gata3 in the early developing otic vesicle. Shown here is that *Fgf10* is specifically affected by levels of Gata3 in the cochlea, and at a later time point than shown previously. *Fgf10* was the most affected by loss of Gata3 compared to the other mRNA levels assessed. *Tecta* is not expressed in *Gata3* positive cells and is necessary for normal formation of the tectorial membrane that covers the organ of Corti. Its levels show significant reduction in the mutant. *Atoh1* continues to be expressed (albeit at a significantly reduced level) even though there is no indication of hair cells or organ of Corti. Genes downstream to Atoh1 are undetectable by qRT-PCR. *Islet1*, *Prox1*, and *Bmp4*, are necessary for prosensory specification and patterning of the organ of Corti. In the absence of Gata3 all of these genes are significantly down-regulated. *Notch*, *Smo*, and *Eya1* all thought to be needed to drive prosensory specification are not altered in the absence of Gata3. *Axin2* is a marker of non-sensory tissue of the growing cochlear duct and is not affected. *Fgfr2b* and *Pax2* are necessary for sensory specification, and are significantly upregulated. The overexpression of these genes is not sufficient to drive sensory development. *p<.01, students t-test. Eight ears were pooled together for one biological replicate. Three biological replicates with technical replicates were assessed for each stage.

We used qRT-PCR to identify potential down-stream genes for *Gata3* in the E16.5 cochlea and quantified their expression changes. We compared gene expression between control and *Foxg1^Cre^: Gata3^f/f^* micro-dissected cochleae (Fig. 4). Care was taken to insure no saccular tissue contaminated the sample. This stage was chosen as it was the earliest time-point the short mutant cochlear duct could be selectively micro-dissected from the remainder very reduced ear. Diffusible factors such as *Bmp4* and *Fgf10* have been proposed to influence the correct topology of cells within the organ of Corti [11], [17], [59]–[61]. In the *Foxg1^Cre^: Gata3^f/f^* cochlea both *Fgf10* and *Bmp4* were significantly down-regulated, although *Fgf10* was down-regulated to a greater extent. This is consistent with other data indicating that Gata3 is upstream to Fgf10 in the early developing otic vesicle [62], [63]. In addition, genes known to be expressed in sensory cell development such as *Islet1*, and *Prox1* were significantly down-regulated. Interestingly, *Atoh1* was significantly down-regulated but not completely absent. However, genes downstream to *Atoh1 (Trilobp, Pou4f3, Nhlh1, Nhlh2, Myo7a)* were not detectable by qRT-PCR. *Eya1* and *Notch1*, factors that are necessary for prosensory specification and differentiation in the inner ear [28], [64], were not altered in the *Gata3* conditional mutant mice. *Pax2*, known to be necessary for normal cochlear development [12], [65], was upregulated along with *Gata2* in the absence of *Gata3*. This upregulation of *Pax2* and *Gata2* is obviously unable to compensate for the lack of Gata3. *Tecta* is a gene known to be an integral player in tectorial membrane development [66]. The reduction in *Tecta* levels is consistent with the lack of tectorial membrane in these mutants. *Axin2* is known to be expressed in the cochlear duct but not in the sensory epithelia and its expression remains unaltered [67].

The loss of Atoh1 downstream gene expression in hair cells without the complete elimination of *Atoh1* expression led us to speculate that Gata3 not only plays a role in specification but possibly also in the differentiation of neurosensory cells of the cochlea. This is consistent with differentiating spiral ganglion neurons and organ of Corti hair cells expressing Gata3 during differentiation. To analyze the timing of recombination of *Gata3* for specification versus differentiation, we utilized another Cre strain (*Pax2-Cre*) which like *Foxg1^Cre^* is expressed in the otic placode, but somewhat later in development [41], [43]. Pax2 is not expressed in the early embryonic caudal hindbrain where loss of Gata3 is proposed to cause embryonic lethality. Previous data indicated that *Pax2-Cre* may be slightly less efficient than *Foxg1^Cre^*, possibly because of a relatively delayed or more inefficient recombination [68], [69].

### *Pax2-Cre* has a delayed recombination of the *Gata3* floxed allele compared to *Foxg1^Cre^*

In order to understand the apparent differential recombination effectiveness previously reported between these two Cre lines [68], [69], we first assessed the ability of *Pax2-Cre* to recombine the *Gata3* floxed allele over time. The level of the *Gata3* exon4 was not significantly altered from control ears until E10.5 (Fig. 1). This is at least two days later than *Foxg1^Cre^*. To further assess why these two Cre lines would have such an obvious difference in recombining the same floxed gene when they have been shown to be expressed in the otic placode at nearly the same time [40], [43], we utilized qRT-PCR to assess Cre levels (Fig. 1). *Gata3^f/f^* inner ear *Foxg1^Cre^* levels were consistently higher than *Pax2-Cre* levels. At E8.5 when *Foxg1^Cre^* had already recombined floxed *Gata3*, *Pax2-Cre* had an eight fold lower level of expression. This lower level of *Pax2-Cre* may account for the two day delayed recombination of *Gata3*.

### *Pax2-Cre: Gata3^f/f^* ears have altered cochlear neurosensory development

**Figure 5:**
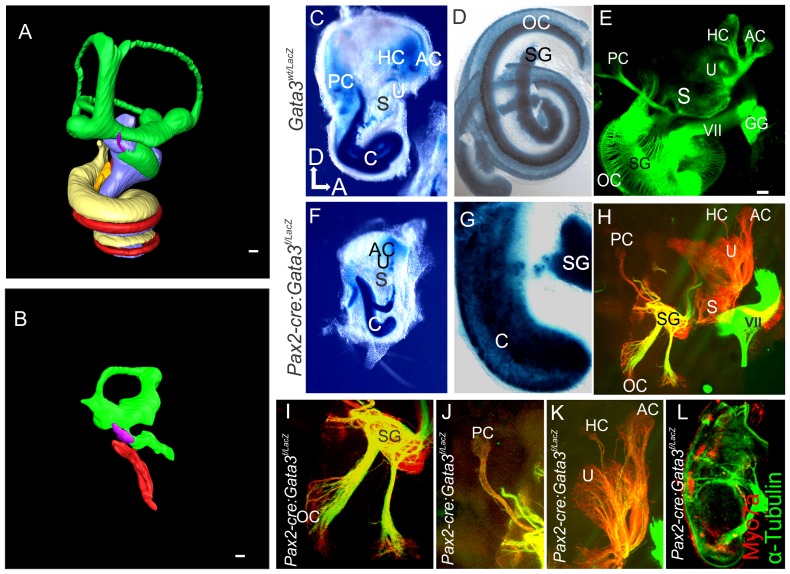
*Pax2-Cre: Gata3^f/f^* ears have altered neurosensory development. **A and B**) 3D reconstruction of a *Pax2-Cre*: *Gata3^f/f^* ear compared to control at P0. Colors correspond to figure 2. **A**) P0 control ear yellow and blue correspond to scala tympani and scala vestibule which are absent in the mutant (**B**). Only the base of the endolymphatic duct has been recsonstructed in both images. **C,D,F,G**) E18.5 Gata3 LacZ expression in *Gata3* heterozygotes with a wildtype allele (C,D) and with a conditional deletion of the second floxed allele with *Pax2-Cre* (F,G). In the mutant there is a reduction in the length of the cochlea. The mutant spiral ganglion (SG) is not adjacent to the cochlear duct, rather all spiral ganglion cells are coalesced in a single area. **E,H-K**) Lipophilic dye tracing as in Fig. 3. In contrast to the *Foxg1^Cre^* mutant spiral ganglion cells are present and project to the cochlea. In contrast to control, (E), innervation to the cochlea is patchy and is targeted to specific areas. Afferent radial fibers project a straight and short distance in the control in contrast to the mutant where the radial fibers project a long distance from a single location of the spiral ganglion neurons to the patches of sensory epithelia. **I-K**) Show higher magnification of innervation to sensory epithelia. **L**) Immunohistochemistry for α-tubulin (green) and Myo7a (red) of mutant cochlear duct. Tubulin staining also shows patchy innervation to the cochlear duct. The remaining innervation targets patches of hair cells that remain along the length of the cochlear duct. Scale bars represent 100 µm. c, cochlea; pc, posterior crista; hc, horizontal crista; ac, anterior crista; VII, facial nerve; gg, geniculate ganglion; u, uturicle; s, saccule; sg, spiral ganglion; oc, organ of Corti.

Assessing the morphology of the *Pax2-Cre: Gata3^f/f^* ear in a 3D reconstruction highlights many differences from a normal control ear (Fig. 5A,B). The vestibular portion contained dorsally a large torus like structure, but had a distinctive saccular out-pouching. The *Pax2-Cre*:*Gata3^f/f^* ear, like that of the *Foxg1^Cre^*:* Gata3^f/f^* mutant had a shortened cochlear duct as is obvious in the 3D reconstruction (Fig. 5A,B). Because the *Pax2-Cre* mutant survives longer than the *Foxg1^Cre^* mutant we were able to assess the development of perilymphatic spaces (scala tympani and scala vestibuli) in the cochlear duct. These spaces were absent in all mutants analyzed. We also analyzed morphology and neurosensory formation using *Gata3^LacZ^* expression. The heterozygous *Gata3^wt/LacZ^* ear contains no obvious morphological or embryonic histologic defects [70]. The *Pax2-Cre* mutant mouse shows a high level of Gata3-LacZ expression in the cochlea as in the control (Fig. 5C,F). Unlike the *Foxg1^Cre^*:*Gata3^f/f^* mutant mice there was frequently a short curvature at the end of the somewhat longer cochlear duct (N = 11/14). One canal crista was present in the anterior part of the ear and a transient formation of a posterior canal crista occasionally occurred (N = 2/7). Unlike the null and *Foxg1^Cre^* mutants there was some formation of spiral ganglion neurons. These few remaining neurons were coalesced into a single location near the base of the shortened cochlear duct, and were not present as a “spiral” ganglion along the length of the cochlea as in the control ear (Fig. 5G-I). The fibers projecting from the spiral ganglia to the cochlear duct formed in distinct bundles and projected in a patchy distribution (Fig. 5H,I). We also assessed the innervation of the *Pax2-Cre*: *Gata3^f/f^* vestibular epithelia using lipophilic dye tracing. Afferent projections were robust to the saccule, utricle, posterior crista, and anterior crista (Fig. 5H,J,K). There were few fibers coursing to the horizontal canal crista (Fig. 5K). Because of the patchy distribution of the spiral ganglion fibers we assessed their distribution with acetylated α-Tubulin and hair cell distribution with Myo7a (Fig. 5L). The Tubulin labeling matched that of the lipophilic dye and Myo7a distribution showed presence of hair cell patches in the cochlear duct. These patches were targeted by the bundles of spiral ganglion neurons.

**Figure 6:**
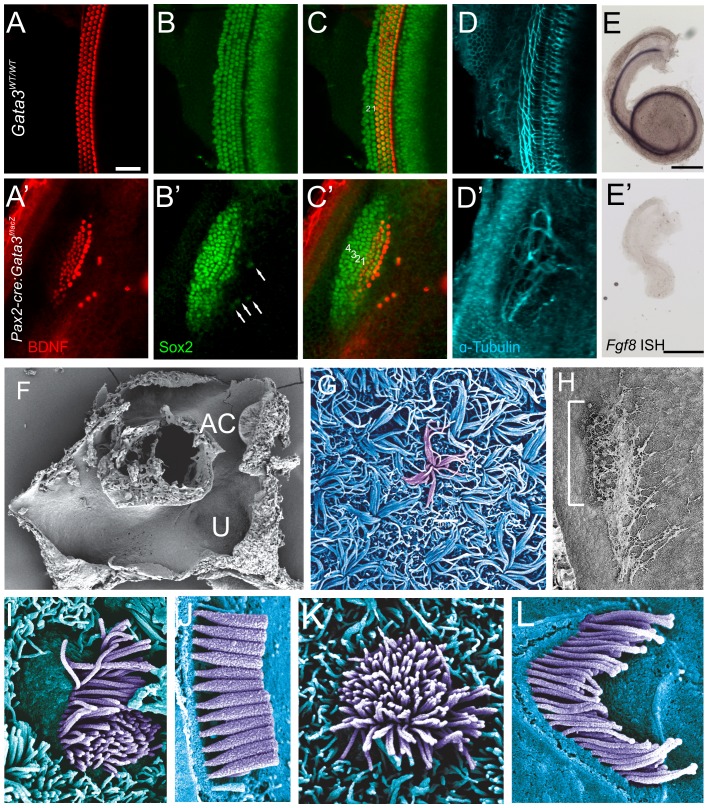
*Pax2-Cre*: *Gata3^f/f^* ears have altered organ of Corti topology. **A-D**) High magnification of organ of Corti showing immunohistochemistry for BDNF (red, hair cells), Sox2 (green, supporting cells), α-tubulin (cyan, neurons). In the mutant patch of sensory epithelia there is one row of inner hair cells and three rows of outer hair cells as in the control. However there are some ectopic BDNF positive cells which are always paired with a Sox2 positive cell (arrows **B**′). A major difference between the mutant and control is where the hair cells are located within the Sox2 expressing domain. In the control there are very few Sox2 expressing cells lateral to the hair cells and a larger portion medially. However, this setup is reversed in the mutant; indicating that there is a shift in the relative topology of hair cells and the Sox2 positive supporting cell domain. The neurons of the mutant are overshooting the hair cells into the ectopic Sox2 domain then looping. **E and E**′) *Fgf8* in situ hybridization in control and mutant cochlea. Even though BDNF staining indicated specification of inner versus outer hair cells, the inner hair cell marker *Fgf8* was absent. **F**) Low magnification SEM of the torus like vestibular structure. The saccular pouch is not shown. **G**) The utricular stereocilia lack connections between each other and are splayed out in a random fashion. Stereocilia from a single hair cell have been false-colored violet. **H**) Low magnification of cochlear duct. The sensory patch apical-basal extent is marked by the white bracket. The abnormal tectorial membrane can be seen as a web like structure that partially covers the sensory patch. There is no spiral limbus in which the tectorial membrane is attached. **I**) A single *Pax2-Cre*: *Gata3^f/f^* hair cell in the location of an inner hair cell. The thickness of the stereocilia is different than in the wt (J) and does not tapper at the bottom. **K**) Hair cell in the location of an outer hair cell. There is no polarity with respect to the stereocilia, and no staircase pattern as in the control (L). All images are of E18.5 cochlea except J and L (P5).

The most striking difference between the *Pax2-Cre*:*Gata3^f/f^* ear and *Foxg1^Cre^*:*Gata3^f/f^* ear was the presence of Myo7a patches within the cochlear duct. We further investigated these sensory patches with other makers. These patches of epithelia contained hair cells supporting cells, and nerve fibers as seen by Bdnf, Sox2, and Tubulin immunochemistry (Fig. 6A-D). Hair cell staining with Bdnf showed 4 rows of hair cells within the sensory patch, with a few ectopic hair cells on the neuronal side of the cochlea (Fig. 6A′). These single ectopic hair cells were each associated with a single Sox2 positive cell (Fig. 6B′C′). The neuronal side of the organ of Corti showed what appeared to be a single row of inner hair cells. Bdnf was more intensely stained in this putative row of inner hair cells, indicating it was different from the other three rows and there may be inner and outer hair cell distinctions in the mutant sensory epithelia. However, Fgf8, a gene known to be expressed in inner hair cells and necessary for pillar cell development [71]–[73], was undetectable in the mutant (Fig. 6E′). In the control mouse the hair cells followed the normal curvature of the cochlea. Although the mutant cochlear duct curved in this same anterior direction there was a mirror image to the curvature to the hair cells. Examination of the control Sox2 expression showed the hair cell domain to be centered within the Sox2 domain. However, Sox2 expression was shifted with respect to the Bdnf positive hair cells and extended further to the abneuronal side of the epithelium (Fig. 6C′). The neuronal projection did not target the hair cells in an orderly fashion, but remained within the Sox2 positive domain, indicating that they can navigate to but not within the sensory patches (Fig. 6D′).

We further investigated the vestibular and cochlear patches of hair-cell-like cells in the *Pax2-Cre*:*Gata3^f/^* ^f^ mutant cochlea using SEM to analyze hair cell morphology (Fig. 6F-L). The vestibular system displayed a torus like structure as indicated by the 3D reconstruction. The anterior canal could easily be distinguished as well as hair cells of the utricle (Fig. 6F). The utricular hair cells were unique in that their stereocilia were not held together properly and laid to the side (Fig. 6G). In the cochlear duct the tectorial membrane of the *Pax2-Cre*:*Gata3^f/f^* cochlea had several irregularities. First, it was not continuous along the length of the cochlear duct, and was loosely associated with a single patch of hair cells, but extended both basally and apically (Fig. 6 H). In addition, the tectorial membrane had several holes or perforations like netting through which the hair cells could be seen. The distribution of patches closely matched the position and size of the Myo7a and Bdnf positive cells seen with immunocytochemistry. As seen with the Bdnf labeling there were apparent inner versus outer hair cell distinctions. The row of hair cells located most neuronally displayed a unique arrangement, of what appeared to be microvilli as they did not show typical stereocilia characteristics, different from the three abneuronal rows. From this we concluded that there was most likely one row of inner and three rows of outer hair cells. The inner hair cell row displayed an orderly polarization with the taller microvilli on the abneuronal portion of the cell (Fig. 6I). In addition the microvilli had no tapering near the cell surface thus lacking a pivoting constriction near their insertion into the hair cells (Fig. 6I,J). The outer hair cells had no apparent polarity with respect to height or distribution of microvilli (Fig. 6K,L).

**Figure 7:**
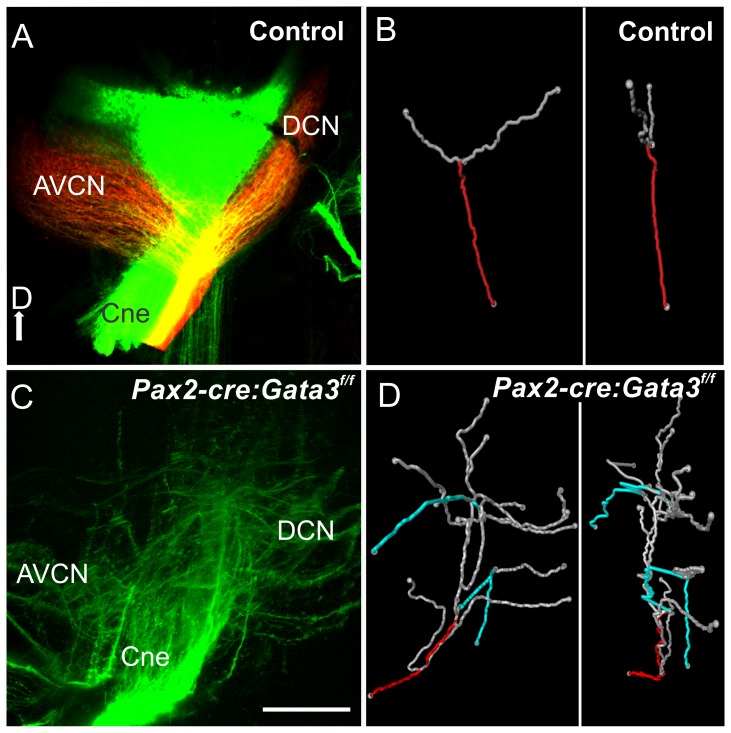
Inner ear central projection. Lipophilic dye was placed into the cochlea of E18.5 mice. In the control mouse red and green dyes were placed into the base and apex. **A and C**) Confocal image of cochlear nerve (Cne) projection into the hindbrain (cochlear nucleus). In the control there is a single bifurcation of each afferent axon to the dorsal cochlear nucleus (DCN) and antero-ventral cochlear nucleus (AVCN). In the mutant the cochlear fibers are bifurcating at several branch points with the terminal fibers looping and misdirected. **B and D**) Amira software was used with the confocal images from **A and C** to trace individual axons. A single axon was reconstructed, and the root of this axon is labeled in red, and its branches in gray. **B**) A representative control axon. Each axon analyzed had a single bifurcation. **D**) In the mutant blue branches are neurites looping back toward the entry point. The single neuron has many bifurcations compared to the control. The right panel of **B and D**) shows the fibers rotated 90 degrees. The control shows the fibers staying within a single plane. The mutant fibers do not show this restriction in 2D space and branch throughout the dorsal-ventral extent of the hindbrain.

Because the spiral ganglion neurons had defects in their peripheral projections we wanted to assess how these neurons project centrally. Lipophilic dye was placed into the inner ear of both mutant and control ears and the cochlear projection was imaged in the cochlear nucleus as whole mounts (Fig 7). The *Pax2-Cre*: *Gata3^f/f^* cochlear nerve correctly targeted the cochlear nucleus, but once the fibers reached this area they were disoriented compared to control (Fig 7). The control fibers entered the hindbrain and had a single bifurcation, forming two divisions to the dorsal cochlear nucleus (DCN) and anteroventral cochlear nucleus (AVCN). The mutant fibers did not seem to have this specificity and directionality. To evaluate what was occurring on an individual neurite level we used Amira software to trace multiple single fibers as they entered the hindbrain, and 3D reconstructed these fibers. The control neurites showed a single bifurcation point, and once entering the hindbrain stayed within a single dorsal-ventral plane. The mutant fibers had multiple bifurcation points and non-specific extensions within the cochlear nucleus. These fibers also projected dorsal-ventrally and did not stay in a single plane, as in the control (Fig. 7B,D).

In summary, the *Pax2-Cre*: *Gata3^f/f^* mutant shows delayed loss of *Gata3* compared to the Foxg1^Cre^ mutant. This results in more vestibular and cochlear development. Most importantly, the delayed recombination of *Gata3* with Pax2-Cre leads to patchy formation of hair cells in the shortened cochlear duct. Those patches of hair cells are innervated by disorganized bundles emanating from disorganized spiral ganglion cells that display a disorganized central projection. Thus, while the delayed loss of *Gata3* allows some neurosensory development of the cochlea, the remaining neurosensory cells apparently need continued expression of Gata3 for normal maturation.

## Discussion

Previous work has demonstrated that complete absence of Gata3 leads to arrest at the otocyst stage with limited morphogenesis and histogenesis [30], [38], [39], [62]. Consistent with these null mutant data, our data from the *Foxg1^Cre^* mutant indicates that without early expression of *Gata3* there is absence of cochlear neurosensory histologic development. In contrast, the *Pax2*-*Cre* conditional deletion data show that delayed deletion of *Gata3* is compatible with some hair cell and spiral ganglion neuron formation. However, with delayed deletion of *Gata3* cochlear neurosensory cells do not undergo normal differentiation. Taking both of these Cre lines into account indicates that early expression of *Gata3* is necessary for neurosensory specification and hair cell differentiation, while delayed deletion of *Gata3* allows for some neurosensory specification with differentiation being aberrant.

### Continued Gata3 expression is required for normal neurosensory development of the cochlea

Our results indicate that in the absence or reduced presence of Gata3, some prosensory cells remain, but are not fully specified and lack full upregulation of *Atoh1* for hair cell differentiation in the cochlea. The lower level of *Atoh1* is striking since other genes known for regulation of *Atoh1* expression, *Jag1* and *Eya1*, are still expressed or even elevated in the cochlear epithelium, such as *Pax2*. This basal level of transcription of *Atoh1* in the mutants may be driven in part by these other factors. The lower level of *Atoh1* is either not sufficient to drive known downstream genes (*Pou4f3*, *Myo7a*, *Nhlh1*, *Nhlh2*, *Triobp*), or *Gata3* is necessary for *Atoh1* function. This is consistent with some bHLH genes in Drosophila requiring the *Gata3* homolog, *pannier*, for both upregulation of these factors and their DNA binding on downstream genes [74]. A survey of transcription factor binding databases showed several potential Gata3 binding sites in several of the genes shown to be disregulated here in the absence of Gata3 including: Pou4f3, Pax2, Sox2, Fgf10, Fgf8, and Atoh1. A DNA binding study needs to be accomplished in the ear as in the trophectoderm [75], T cells [76], and avian vestibule [77] to verify that these genes are directly regulated by Gata3.

With a slight delay in *Gata3* recombination (*Pax2-Cre*) we were able to partially assess hair cell and spiral ganglion differentiation in the absence of Gata3. While spiral ganglion neurons are completely absent in the *Foxg1^Cre^* ear few spiral ganglion neurons form in the *Pax2-Cre* ear. However, these spiral ganglion neurons do not differentiate properly as is evident by the aberrantly projecting to the sensory patches in the cochlear duct and the cochlear nucleus. There is a curious resemblance between this mutant and the conditional knockout of Neurod1 utilizing the same Cre line [71]. Both the *Neurod1* and *Gata3* mutants have reduced numbers of spiral ganglion neurons, spiral ganglia cell bodies incorrectly migrate away from the ear towards the hindbrain, and have incorrect patterning within the cochlear nucleus. Interestingly both of these mutants have spiral ganglia that project to the correct regions (organ of Corti and cochlear nucleus), but, once there, do not correctly target appropriate cell types. In addition, timing of *Gata3* expression has indicated a role in regulating the Insulin-like Growth Factor (IGF) pathway [78]. IGF mouse mutants have been shown to have reduced numbers of spiral ganglion neurons as well as incorrect projections within the organ of Corti [79], [80], consistent with Gata3\IGF interaction. Remarkably the effect of Gata3 on prosensory specification in the organ of Corti must occur before E10.5, the time recombination is apparently complete with *Pax2-Cre*). This is interesting since hair cells in the cochlea do not exit the cell cycle before E11.5 [21], [81] and do not initiate differentiation in terms of Atoh1 expression until E13.5 [21], [72]. Thus, although prosensory marker genes such as Sox2 [6] may highlight the cochlear anlage between E10.5 and E14.5 and a substantial amount of proliferation takes place during this time [82], these cells may already be specified to be at least prosensory and require Gata3 together with other factors such as Eya1 [28], Sox2 [10] and Pax2 [12] for this identity.

*Atoh1* has been shown to be an essential factor for hair cell development in the ear [18] and precise levels and timing of *Atoh1* are essential for hair cell development and viability [16], [17]. Indeed uncontrolled upregulation of *Atoh1* does not result in long lasting and physiologically functional auditory hair cells [23], [24], [83]. While *Sox2* and *Eya1/Six1* have been shown to be necessary for bHLH gene upregulation in the ear, how many other factors are needed in addition remains unclear. Until now no factor has been shown to be exclusively necessary for cochlear and not vestibular hair cells, with the former being known to be more susceptible to a number of genetic and pharmacological insults. We provide here evidence that *Gata3* may enable *Atoh1* mediated hair cell differentiation in particular in the cochlea. This conclusion is consistent with recent data showing that *Gata3* enhances the ability of *Atoh1* to upregulate downstream genes [84]. Our data show that other factors believed to be essential for neurosensory differentiation (*Eya1*, *Pax2*, *Jag1*), which remain expressed in the *Foxg1^Cre^:Gata3^f/f^* cochlea, are unable to drive *Atoh1* expression or neurosensory differentiation in the cochlea in the absence of *Gata3*. Our data therefore establish Gata3 as an essential player in the emerging molecular network needed for neurosensory development.

### Gata3 is needed for spiral limbus and tectorial membrane formation

The lack of a spiral limbus, the formation of an incomplete tectorial membrane and formation of only microvilli on hair cells in the Pax2-Cre: *Gata3^f/f^* cochlea suggests that sustained and profound expression of *Gata3* is needed for normal development of these structures. The down-regulation of *Tecta* in the *Foxg1^Cre^* mutant mouse nicely fits into this phenotype as *Tecta* is necessary for tectorial membrane formation [85] which is absent (Foxg1^Cre^) or severely reduced (Pax2-Cre) in *Gata3* mutants. The mal-development of hair cell microvilli in the Pax2-Cre:*Gata3^f/f^* cochlea suggests that Gata3 is playing a role not only in hair cell specification but also in hair cell stereocilia differentiation. An assessment of genes selectively down-regulated in these hair cells would be beneficial in elucidating which genes are deregulated in the absence of Gata3 during differentiation. Among those genes could be many microRNA regulated genes as the overall development of hair cells is reminiscent of Dicer1 null mice cochlear hair cells [69].

### Gata3 is needed for semicircular canal formation

Knocking out *Gata3* expression early in ear development in Foxg1^Cre^: *Gata3^f/f^* mice abolishes cochlear neurosensory formation. However, the saccule, utricle and a single anterior canal crista form hair cells and receive afferent and efferent innervation, indicating that *Gata3* is less critical in vestibular neurosensory development compared to the cochlea. These ears develop a very short cochlear duct, saccular and utricular recesses, but lack semicircular canals. The lack of canal formation could be caused by lack of canal crista known to be necessary for canal growth [11], [86], [87] or due to Gata3 which is expressed in the canal plate [30], [31]. We show that while canal cristae may be necessary for canal formation, they are not sufficient in the absence of Gata3. Recent models suggesting interplay of Fgfs and Bmps [88] for canal growth need to be modified to include Gata3. Our data align for the first time the formation of the two vertical canals to that of the horizontal canal. In the latter it was shown that a crista can form without causing canal formation [82], [89] and that a canal can form in the absence of a crista [42]. Further work is needed to understand how absence of Gata3 disrupts vertical canal growth in the presence of canal cristae beyond the obvious candidates of reduction of Fgf10 [11], [62]. This is particularly important in the Pax2-Cre:*Gata3^f/f^* mutant where a single toral structure forms despite the presence of up to three cristae. Formation of a short cochlear duct is consistent with findings in other vertebrates that form outgrowth of such ducts without neurosensory formation [90].

### Olivocochlear efferents migrate normally without a target

Interestingly, we were able to label the olivo-cochlear efferents in the hindbrain in both mutant lines which migrate their soma into the correct periolivary location [49]. While these efferents project out of the hindbrain and can therefore be filled by peripheral dye application, they do not innervate their correct peripheral target, the organ of Corti in the Foxg1^Cre^ mutant. We find efferent fibers to vestibular epithelia and presumably some may be olivocochlear efferents which may also project with the facial nerve as previously reported [38], [55]. While inner ear efferents express *Gata3* [30] *Foxg1^Cre^* and Pax2-Cre are not expressed in these populations, indicating that this is not a cell autonomous effect. Thus with the loss of their original target vestibular and cochlear efferents are able to segregate, and cochlear efferents are able to survive at least a short time without proper targets.

**Figure 8:**
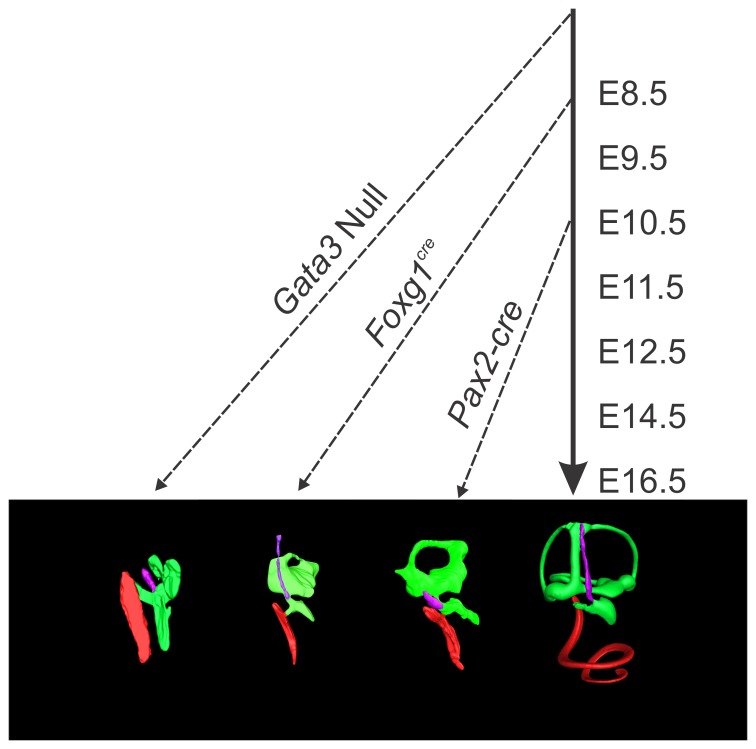
Timing of *Gata3* loss reveals different phenotypes. The loss of *Gata3* during different stages of development reveals changing roles of *Gata3* in cochlear neurosensory development. In this diagram the solid arrow indicates normal embryonic progression while the dashed arrows indicate the aproximate embryonic age of *Gata3* loss from the ear and subsequent altered development. In the *Gata3* null mutant [38] the inner ear was shown to contain only a single patch of sensory epithelia (saccule) and corresponding afferent neurons. The cochlear duct was completely devoid of all neurosensory cell types. In the *Foxg1^Cre^* conditional *Gata3* mutant there is formation of saccule, utricule, and anterior canal neurosensory cell types. The cochlear sensory epithelia is not properly specified, some prosensory genes remain, and many sensory differentiation genes are absent or downregulated. Only in the *Pax2-Cre* mutant where recombination occurs later than the other two indicated muants (between E8.5 and E10.5) are some cochlear neurosensory cells present, but this later loss of *Gata3* results in improper differentiation of these cell types.

In summary, our data suggest that Gata3 is essential for normal neurosensory development of the cochlea that acts at multiple levels to ensure differentiation of the organ of Corti and its proper innervation and associated structures such as the spiral limbus and tectorial membrane. *Gata3* expression in the forming semicircular canals plays an essential role in translating canal crista signals into proper canal growth. Sustained Gata3 expression is not only needed to initiate development of neurosensory cells but its reduction affects the normal differentiation leading to unusual hair cell formation and aberrant spiral ganglion cell projections. Cochlear development thus depends at multiple levels on the continued presence of Gata3 and future work is now needed to elucidate how Gata3 co-operates with an emerging network of other factors to achieve these multiple functions (Fig. 8).

Note added in proof. While our manuscript was in production a related article was published further detailing the role of Gata3 in spiral ganglion neurons [91]. The results complement our findings that loss of *Gata3* results in aberrant spiral ganglion neurite outgrowth to the organ of Corti.

## Supporting Information

Table S1 **This table lists sequences and relevant data for all qPCR primers used.**
(XLSX)
